# Depressive Symptoms and Resilience among Pregnant Adolescents: A Case-Control Study

**DOI:** 10.1155/2010/952493

**Published:** 2011-02-21

**Authors:** Danny Salazar-Pousada, Dalton Arroyo, Luis Hidalgo, Faustino R. Pérez-López, Peter Chedraui

**Affiliations:** ^1^High Risk Pregnancy Labor and Delivery Unit, Enrique C. Sotomayor Obstetrics and Gynecology, Hospital Guayaquil, 196 Guayaquil, Ecuador; ^2^Facultad de Ciencias Médicas, Universidad Católica de Santiago de Guayaquil, 196 Guayaquil, Ecuador; ^3^Department of Obstetrics and Gynaecology, Hospital Clínico de Zaragoza, Facultad de Medicina, Universidad de Zaragoza, 50009 Zaragoza, Spain

## Abstract

*Background*. Data regarding depression and resilience among adolescents is still lacking. *Objective*. To assess depressive symptoms and resilience among pregnant adolescents. *Method*. Depressive symptoms and resilience were assessed using two validated inventories, the 10-item Center for Epidemiologic Studies Short Depression Scale (CESD-10) and the 14-item Wagnild and Young Resilience Scale (RS), respectively. A case-control approach was used to compare differences between adolescents and adults. *Results*. A total of 302 pregnant women were enrolled in the study, 151 assigned to each group. Overall, 56.6% of gravids presented total CESD-10 scores 10 or more indicating depressed mood. Despite this, total CESD-10 scores and depressed mood rate did not differ among studied groups. Adolescents did however display lower resilience reflected by lower total RS scores and a higher rate of scores below the calculated median (*P* < .05). Logistic regression analysis could not establish any risk factor for depressed mood among studied subjects; however, having an adolescent partner (OR, 2.0 CI 95% 1.06–4.0, *P* = .03) and a preterm delivery (OR, 3.0 CI 95% 1.43–6.55, *P* = .004) related to a higher risk for lower resilience. *Conclusion*. In light of the findings of the present study, programs oriented at giving adolescents support before, during, and after pregnancy should be encouraged.

## 1. Introduction

Adolescence is a time of physical changes, psychological maturation, and social value acquisition. Teenagers face many challenges and stressful situations related to educational commitment, social behaviour, sexual development, familial conflicts, economical problems, and substance abuse. These factors may certainly modulate personality and individual behaviour. Recent reports indicate that adolescents are initiating sexuality at an earlier age than in the past; in many cases contraceptive measures are not being used [1]. Pregnancies among adolescents are considered as a complication, as they favour education interruption, poor present and future health, higher rates of poverty, problems for present and future children, among other negative outcomes [2]. 

Adolescents display emotional responses toward an undesired pregnancy, presenting higher school dropout rates, social punishment, and segregation [1, 3]. Anxiety, stress, and depression are among the most frequently encountered reactions toward an unexpected pregnancy [4]; however, there is limited information regarding this reaction among adolescents as compared to older pregnant women [5]. Resilience, on the other hand, has been defined as the capacity that allows an individual to prevent, minimize, or overcome damage imposed by life adversity [6, 7]. It is a measure on how individuals cope, overcome, or even become positively strengthened by changes and challenges. Resilience is pivotal for adolescents to mature in healthy ways, including sexual health and well-being maintenance. Adolescents face many difficulties and stressful situations: personal achievements (education and work), sexual development, family conflicts, sociocultural issues, substance use/abuse, antisocial behaviour, among others. Individuals with high resilience are less likely to engage in risky behaviors [7]. Furthermore, complex relationships exist between depressive symptoms and resilience in adolescents. 

Despite the fact that resilience is important for adolescent personality maturation, up-to-date, studies assessing depressive symptoms, and resilience specifically in pregnant adolescents are still lacking. The present study aimed at assessing depressive symptoms and resilience among pregnant adolescents. It was hypothesized that prevalence of depressed mood would be greater and resilience lower among adolescents as compared to controls aged 20 to 30 years. Studies addressing resilience during pregnancy are scarce [8–10]. It has been assessed during pregnancy in various stressful situations: after ultrasound consultation for fetal malformations [8] and after exposure to a hurricane [9]. Another report examined resilience factors (maturity, self-esteem, and mother-grandmother relationships) shortly after delivery and parenting behavior at 6 months although no specific resilience measuring tool was used [10]. Despite the fact that resilience is important for adolescent personality maturation, up-to-date, studies assessing depressive symptoms and resilience specifically in pregnant adolescents are still lacking. The present study aimed at assessing depressive symptoms and resilience among pregnant adolescents. It was hypothesized that prevalence of depressed mood would be greater and resilience lower among adolescents as compared to controls aged 20 to 30 years.

## 2. Methods

### 2.1. Study Design and Participants

This case-control study was carried out from 1 February 2010 to 30 April 2010 in the Labor and Delivery Unit of the Enrique C. Sotomayor Obstetrics and Gynecology Hospital of Guayaquil, Ecuador after approval of the institution's Scientific, Research and Ethics Committee. Nulliparous women aged 19 or less, delivering at this facility a live, single child of more than 20 weeks in the working shifts of one of the authors (D.S-P; 12 hrs every 24 hrs) were considered eligible to be included as cases (adolescents). Those with an abortion and/or still births were excluded. Each teenager was surveyed in the first postpartum hour with a structured questionnaire containing general maternal data, the 10-item Center for Epidemiologic Studies Short Depression Scale (CESD-10) and the 14 item Wagnild and Young Resilience Scale (RS) [11–13]. Survey was pretested on several teenagers prior to the initiation of the study. Maternal and neonatal outcome data were directly assessed from the medical records of each subject. Delivery of a nulliparous woman (20 to 30 years) right after each indexed case was selected as the corresponding control. Controls were identified through the delivery log book and surveyed in the same fashion as cases. Participants were informed of the study, its objectives, and confidentiality and their full right to discontinue or refuse participation. Interviews were maintained anonymous and consent obtained orally. 

The Enrique C. Sotomayor Obstetrics and Gynecology Hospital of Guayaquil, Ecuador, performs more than 30,000 deliveries per year, attending basically gravid women of low socioeconomic income of Guayaquil, the major coastal city of Ecuador with a population above 3 million inhabitants. Approximately 25% of annual deliveries correspond to those aged 19 or less [14].

### 2.2. Center for Epidemiologic Studies Short Depression Scale (CESD-10)

The CESD-10 is a 10-item questionnaire assessing how individuals feel during the past week. This is a short version of the 20-item CESD tool. Each item can be graded according to a Likert scale: rarely or none of the time, <1 day (0 points); some or a little of the time, 1-2 days (1 point); occasionally or a moderate amount of time, 3-4 days (2 points); and all the time, 5 to 7 days (3 points). Items 5 and 8 are scored inversely. Final score is the sum of the 10 graded items with scores 10 or greater considered as depressed mood [11, 12].

### 2.3. The Resilience Scale (RS)

The 14-item Wagnild and Young Resilience Scale (RS) was used to assess resilience status. This scale was constructed after interviewing resilient subjects and hence is an accurate tool for studying resilience. It is a Likert type scale used in various age groups and different conditions. Each item can be graded from “1” (strongly disagree) to “7” (strongly agree) [13]. Graded items are summed up to provide a total score. Although no cut-off value for abnormality is available, lower scores indicate less resilience.

### 2.4. Sample Size Calculation

It has been reported that 10–20% of all pregnancies may present depressed mood [5], with a 46% found among teens [15]. Hence, assuming a 15% prevalence of depressive symptoms in the control group, a sample size of 125 subjects per group (cases and controls) was calculated in order to detect a 2.5 increased risk of depressed mood among adolescents with an 80% power and a 95% confidence level.

### 2.5. Statistical Analysis

Analysis was performed using statistical packages: SPSS (Version 10.0 for Windows, SPSS, Chicago, IL, USA) and EPI-INFO 6.04 (Centers for Disease Control and Prevention, Atlanta, GA, USA/World Health Organization, Geneva, Switzerland). Data are presented as mean ± standard deviations, medians, percentages, odds ratios (OR), and confidence intervals (CI). Kolmogorov Smirnov's test was used to determine normality of data distribution. According to this, continuous nonparametric paired data was compared with the Wilcoxon signed-rank test. Percentages were compared with the chi-square or Fisher's exact test. Logistic regression analysis was used to determine risk factors related to depressed mood (Total CESD-10 scores 10 or more) and lower resilience (Lower total RS scores). Variables included in the regression model were those related to the: *mother *(age, habits, marital status, place of residency, adequacy of prenatal care), *pregnancy* (maternal and neonatal outcome data), and the *partner* (age and habits). Interactions were also considered during regression model construction. A *P* value of <.05 was considered as statistically significant.

## 3. Results

During the study period, there were a total of 1,138 live singleton deliveries. Of these, 151 were nulliparous adolescents (13.3%). 151 nulliparous controls were selected for all cases giving a total of 302 gravid women who were surveyed. General demographic data of studied women are shown on Table 1. Adolescents had a nonmarried status and were studying in a higher rate than their counterparts. Partner age and grandmother's age at first child were significantly lower among adolescents. Gestational age at first antenatal visit was significantly higher among adolescents. Cesarean section rate among teenagers was significantly lower than controls (34.4% versus 47.7%, *P* = .01). Women displaying depressed mood (*n* = 171/302) had more cesarean section (41.1% versus 36.6%, *P* = .44). When depressed women were stratified as adolescents and adults, adolescents presented a similar cesarean rate (35.4% versus 33.3%) whereas adults a higher one (52.2% versus 40.7%) as compared to nondepressed ones; however, these comparison, were not found to be statistically significant. No other differences in maternal and neonatal data were observed among studied cases and controls (Table 2). 

Total CESD-10 and RS scores found among studied women are depicted on Table 3. A 56.6% of all studied women presented CESD-10 total scores of 10 or more indicating depressed mood. Mean total CESD-10 scores and depressed mood rate did not differ among studied groups. Contrary to this, adolescents displayed lower RS total scores (indicating less resilience) and a higher rate of scores below the calculated median (*P* < .05). Logistic regression analysis (even after included several interactions) could not establish any risk factor for depressed mood among studied subjects; however, having an adolescent partner (OR, 2.0 CI 95% 1.06–4.0, *P* = .03) and a preterm delivery (OR, 3.0 CI 95% 1.43–6.55, *P* = .004) related to a higher risk for lower resilience.

## 4. Discussion

Adolescent pregnancies are increasing worldwide in relation to several biological, social, and personal factors. They are unintended in the majority of cases and create negative feelings both in adolescents (mother and progenitor) and their families. Although pregnant teenagers have similar obstetrical issues as older gravids, additional risks may appear when socioeconomical factors are taken into account [3, 16]. Sociodemographical characteristics of pregnant women (teenagers and non teens) of this series reflect those of the low income population of the Ecuadorian coast which are cared for at the Sotomayor Hospital. There were no significant differences between pregnant adolescents and young adult women in parameters such as lifestyle habits, rural residency, and prenatal care. Despite this, it is worthy to mention that women in the control group (also nulliparous) were in fact also young (mean 23 years). This maybe due to the fact that low income women cared for at Sotomayor initiate parity young, hence by 30 women already have 2 o 3 siblings. Both groups currently lived together with their partner in a similar rate, yet teenagers had a nonmarried status in a higher rate than adults. Although a significant higher proportion of adolescents were studying at the moment of the survey, this rate may be seen as low, moreover if the rest were working, doing nothing, or working and studying. General fertility rate in Ecuador is among the highest of Latin America and has increased from 84 to 100 per 1,000 in 2004. A 20% of women get pregnant before age 20 and 43% of illiterate adolescents have been pregnant as compared to 11% of those with higher education [17]. Although the number of prenatal visits was similar in studied groups, adolescents of the present series initiated some 2-3 weeks later as compared to young adults. These data could be considered as expectable in adolescents and related to the “*surprise*” of being pregnant and the difficulties of assuming pregnancy and obtaining appropriate care. 

The risk of pregnancy among adolescents is related to family structure, education, and care. Risk increases with a previous teen pregnancy, lower partner age, and having mothers who were also pregnants adolescents. Partner and grandmother's age at first child was significantly lower among adolescents of our series, which is in correlation with other reports [18]. 

Perinatal outcome among adolescents of this study was similar to adults in terms of preterm birth rate, small-for-gestational-age (%), Apgar scores, and neonatal weight. These results support those of others [3, 19]. Reports indicate that adolescents have both shorter first and second stages of labor as compared to the general obstetrical population [20]. There is no clear explanation for this finding; however, it could be related to the way women experience the onset of their labor. A large proportion of these experiences bear no resemblance to the classical diagnosis of labor and most are unrelated to labor duration [21]. Although progression of labor was not specifically analyzed in this series, vaginal delivery rate was higher among adolescents supporting the findings of others [20]. Progression of labor may depend on factors such as maternal and fetal weight, ethnics, and the type of used analgesia. Overall cesarean section rate was high in our series as compared to results from other latitudes [22, 23]. Explanation to this may rely on the fact that Sotomayor hospital is a major referral obstetrical institution for low income women of a vast population of the Ecuadorian coast with high rates of inadequate prenatal care and medical/obstetrical complications. A lower cesarean section rate was found among our adolescents which correlates with other reports [3, 16, 22].

Depressive episodes may affect adolescents in up to 15% of cases, being more frequent among women with negative cognitions, interpersonal conflicts, low social support, and stressful life events [24]. Pregnant adolescents suffer depression, anxiety, frustration, and aggression in a higher rate than gravid adults [25]. Depression prevalence is much higher among pregnant teens than in adults, with rates varying from 16 to 50% [5, 15, 24, 25]. This wide prevalence range may reflect differences in sample composition (educational level, rural versus urban residency, minority groups, social support) and how depression is diagnosed. In our study, depressed mood and resilience were categorized using two validated tools. Depressive symptoms were assessed with the CESD-10, a tool widely used for depression research in the general population [12]. Other tools used to assess depressive mood include the Beck Depression Inventory, the Edinburgh Postnatal Depression Scale, the World Health Organization's Composite International Diagnostic Interview Short Form, among others [24]. The CESD-10 measures only current symptoms and reasonably identifies clinically depressed from nondepressed subjects [11]; moreover, the CESD-10 has shown high sensitivity, specificity, and positive predictive values [12]. 

Previous studies addressing depressive symptoms in pregnancy have been performed during prenatal consultations [26]. Depressive symptoms increase during the last trimester of pregnancy [27]. Positive depressive score for the 20-item CESD tool was more frequently found at mid-pregnancy among teens (46%) and disadvantaged American women (47%) [15]. Using the CESD-10, it has been reported that one out of five women in the antepartum period presents depressed states. This has been related to younger age, substance use (cigarette or alcohol), and having a past and current obstetrical or medical complication [28]. Using the CESD-10 tool, the present series found a similar rate of depressed mood among studied groups. Logistic regression analysis could not identify any single risk factor for depressed mood. Our findings suggest that factors explaining depressed mood in both groups must be similar and most likely not related to age yet to other conditions such as poverty, cultural, and risk behaviours seen in this specific low income population. Other factors related to depressed mood during pregnancy include cigarette, alcohol, or drug use [29] which were not present in the studied population. 

Although decreased fetal growth has been observed in low income women who present depressive symptoms [30], the effect of psychosocial factors (i.e., depression, anxiety, stress, and low self-esteem) on infant birthweight and gestation duration in this population is still controversial [31]. A recent report found that depressed adolescents with suicidal ideation or attempts (as compared to those without) delivered babies with lower birthweight [5]. Perinatal outcome in our series did not differ even after stratifying for the presence of depressed mood. CESD-10 scores indicative of depressed mood have been associated to higher assisted vaginal deliveries and cesarean section rates [32]. This trend was not observed in the present series. 

Resilience is a complex set of values that allows a person to withstand many of the negative effects of adversity. Therefore, a resilient individual can cope with adversity. Resilience is central for adolescents to develop and mature in healthy ways, including sexual health and well-being maintenance [6, 7]. Adolescence, the family, and the community seem to modulate resilience. In addition, low self-esteem, unplanned pregnancies, sexually transmitted infections, drug misuse, and lack of family care and guidance may negatively influence resilience [7]. Positive emotions assist high-resilient individuals in their ability to effectively overcome daily stress [33]. Assessing all the components of resilience is not easy. While many tools assess indirect measures of resilience such as self-esteem, sense of coherence, or school adjustment; others include a large number of measures or identify a particular subsample, making assessment difficult in the clinical setting [34]. The resilience tool used in the present series has shown to be appropriate in different situations, display high measures of consistency and correlation with life satisfaction scales [6, 35], and most importantly be useful among adolescents.

Studies assessing resilience during pregnancy are scarce [8–10] and have mainly focused on anxiety/depressive components using nonspecific tools. For instance, one study measured level of anxiety rather than resilience after ultrasound consultation in uncomplicated gravids with different risks of fetal abnormalities [8]. A second report assessed resilience in a cohort of pregnant women exposed to a hurricane. Resilience was based on an interview performed at delivery and 8 weeks later using the Edinburgh depression Scale and the Post-Traumatic Stress Checklist (nonspecific resilience measuring tools) as an indirect measure of mental health resilience after the hurricane experience during pregnancy [9]. The authors concluded that some women were resilient from depression and posttraumatic stress. A third paper examined resilience factors (maturity, self-esteem, and mother-grandmother relationships) shortly after delivery and parenting behavior at 6 months in a African-American adolescent cohort. Despite this no specific resilience measuring tool was used [10]. Our approach aimed at specifically assessing resilience in a sociodemographically homogeneous gravid population (adolescents versus adults) attending a particular healthcare system. To the best of our knowledge, it is perhaps the first to assess resilience in a case, control fashion (adolescents versus controls) at the time of delivery and most of all using a specific validated resilience tool. As compared to adults, our adolescents displayed lower total RS scores indicating less resilience in which having an adolescent partner and delivering preterm were related risk factors. Individual characteristics of teen mothers and positive family support increase their resilience [10]. Our results indicate that there is a need to develop clinical and emotional support programs for pregnant adolescents to strengthen resilience and improve emotional, mental and social capacities to overcome adversity. More research is warranted in this regard. 

A high false positive rate for depressed mood can be found when the CESD tool is used [36]. This could be the case in our study. Although this may be seen as a limitation, it could, on the other hand, reflect a highly prevalent problem characteristic of our low income women. Risk factors for depression in our obstetrical population need to be further addressed; moreover, panic disorders, domestic violence or the presence of several comorbidities, determinants of psychosocial stress during pregnancy, were not explored [37]. We recognize that timing of the survey may also be seen as a limitation. Nevertheless, research was carried in the best possible Ecuadorian conditions considering the fact that our hospital has a high delivery rate of low income women admitted for labor without prenatal care and in many cases discharged 24 or less hours. Followup under these conditions is very difficult and sometimes impossible. 

Despite outlined limitations, important to mention is that this indeed maybe the first case-control study to concomitantly explore depressive symptoms and resilience in a low income pregnant series. The CESD-10 and the RS are easy to use tools and provide a rapid snapshot of the situation. More research in our population is needed to identify risk factors for depressed mood during pregnancy. 

In conclusion, although prevalence of depressive symptoms was similar among studied groups, overall rate found in this series was two times that reported in the literature using the same tool (CESD-10). Adolescents displayed a lower level of resilience when compared to young adult gravids. Future research should aim at measuring resilience in adolescents some time after delivery of their first child in order to quantify their coping capacity and its impact on maternal fetal health. Our results are indicative that social support should be provided throughout pregnancy in order to increase resilience in our adolescent population. Programs need to be designed specifically for our cultural setting (i.e., include the partner and relatives). Positive adaptation to pregnancy—and support—will increase social competence which in turn will aid overcoming the difficult task of becoming a mother. Pregnant adolescents need help to manage negative feelings and communicate their needs to adults and institutions.

##  Conflict of Interests

The authors declare no conflict of interests.

## Figures and Tables

**Table 1:** General demographic characteristics of studied women.

Maternal data	All *n* = 302	Adolescents *n* = 151	Nonadolescents *n* = 151	*P* value*
Age (years)	20.0 ± 3.8	17.2 ± 1.4	23.0 ± 3.0	.001
Non married status (%)	187 (61.9)	103 (68.2)	84 (55.6)	.02
Currently living together (%)	236 (78.1)	116 (76.8)	120 (79.5)	.57
Sometime smoked during pregnancy (%)	1 (0.3)	1 (0.7)	0 (0.0)	.98
Sometime alcohol consumption during pregnancy (%)	15 (5.0)	5 (3.3)	10 (6.6)	.18
Rural residency (%)	86 (28.5)	45 (29.8)	41 (27.1)	.61
Before becoming pregnant				
Was studying	137 (45.4)	102 (67.5)	35 (23.1)	.001
Was working	80 (26.5)	18 (11.9)	62 (41.1)	.001
Nothing	65 (21.5)	24 (15.9)	41 (27.2)	.01
Working and studying	20 (6.6)	7 (4.7)	13 (8.6)	.16
Was performing family planning method before becoming pregnant	47 (15.6)	25 (16.6)	22 (14.6)	.63
Age of grandmother's first child	18.9 ± 3.9	18.7 ± 3.7	19.0 ± 4.0	.001
Number of prenatal visits	7.0 ± 2.6	6.9 ± 2.9	7.0 ± 2.3	.29
Less than 5 prenatal visits	42 (13.9)	22 (14.6)	20 (13.2)	.73
Gestational age at first visit	9.9 ± 6.9	11.1 ± 7.5	8.7 ± 6.2	.001

Partner data				
Age (years)	24.4 ± 5.7	22.0 ± 4.0	26.8 ± 6.0	.001
Current smoking habit	24 (7.9)	16 (10.6)	8 (5.3)	.08
Alcohol consumption	79 (26.2)	39 (25.8)	40 (26.5)	.89
Currently employed	254 (84.1)	124 (82.1)	130 (86.0)	.34

Data are presented as mean ± standard deviations and percentages (%); *P value after comparing cases and controls with Wilcoxon signed-rank test, chi-square test or Fisher's exact test.

**Table 2:** Maternal and neonatal outcome data.

Maternal outcome	All *n* = 302	Adolescents *n* = 151	Non Adolescents *n* = 151	*P* value*
Obstetrical complication (ante/intrapartum) (%)	152 (50.3)	69 (45.7)	83 (55.0)	.10
Vaginal delivery (%)	178 (58.9)	99 (65.6)	79 (52.3)	.01
Cesarean section (%)	124 (41.1)	52 (34.4)	72 (47.7)	.01
Intrapartum meconium staining (%)	13 (4.3)	4 (2.6)	9 (6.0)	.15

Neonatal outcome				
Gestation age at birth (weeks)	38.7 ± 2.0	38.6 ± 1.8	38.8 ± 2.1	.16
Neonatal weight (g)	2,897.0 ± 556.4	2,874.9 ± 518.0	2919.0 ± 593.2	.26
Preterm birth (%)	38 (12.6)	22 (14.7)	16 (10.6)	.29
Small-for gestational-age (%)	62 (20.5)	32 (21.2)	30 (19.9)	.77
Apgar score <7 at 1st min (%)	17 (5.6)	8 (5.3)	9 (6.0)	.80
Apgar score <7 at 5th min (%)	8 (2.6)	6 (4.0)	8 (5.3)	.58
Neonatal complication (%)	39 (12.9)	15 (9.9)	24 (15.9)	.12

*P value after comparing cases and controls with chi-square test or the Wilcoxon signed-rank test.

**Table 3:** Total CESD-10 and RS scores among studied women.

Parameters	All *n* = 302	Adolescents *n* = 151	Nonadolescents *n* = 151	*P* value*
Total CESD-10 score	10.9 ± 5.8	10.5 ± 5.9	11.2 ± 5.7	.31
Depressed mood (%)	171 (56.6)	79 (52.3)	92 (60.9)	.13
Total RS score	80.7 ± 10.5	79.3 ± 10.3	82.0 ± 10.5	.002
Total RS score <82 (median) (%)	141 (46.7)	79 (52.3)	62 (41.1)	.04

*P value after comparing cases and controls with Wilcoxon signed-rank test or the chi-square test.
